# ‘What are you hiding from me?’ A qualitative study exploring health consumer attitudes and experiences regarding the patient‐led recording of a hospital clinical encounter

**DOI:** 10.1111/hex.13617

**Published:** 2022-10-13

**Authors:** Laura Ryan, Kelly A. Weir, Jessica Maskell, Lily Bevan, Robyne Le Brocque

**Affiliations:** ^1^ Allied Health Research, Gold Coast Health Gold Coast University Hospital Southport Queensland Australia; ^2^ Menzies Health Institute Queensland Griffith University Southport Queensland Australia; ^3^ Social Work Services Gold Coast Health Southport Queensland Australia; ^4^ School of Nursing, Midwifery, and Social Work The University of Queensland St Lucia Australia Queensland; ^5^ Gold Coast Health Consumer Advisory Group Gold Coast Health Southport Queensland Australia

**Keywords:** mobile interventions, professional–patient relations, Smartphones

## Abstract

**Objective:**

Health consumers (patients, their family, friends and carers) are frequently using their smartphones to record hospital clinical encounters. However, there is limited research which has explored the social interaction surrounding this behaviour. Understanding the consumer perspective is key to informing policy and practice. This study explored consumer attitudes and experiences regarding patient‐led recordings.

**Methods:**

Semistructured interviews were undertaken with 20 hospital consumers. Participants were recruited via advertising, posters and invitation letters. Interviews were digitally recorded and transcribed. Data were analysed using thematic analysis.

**Findings:**

Four main themes were identified relating to participant perspectives of patient‐led recordings: (1) consumers viewed clinician consent as important, although they reported different experiences of the consent process, (2) consumers indicated that a clinician refusing the recording had the potential to undermine the consumer–clinician relationship, (3) consumers were both uninformed and misinformed regarding relevant policy and legislation and (4) consumers expressed a number of expectations regarding their rights to record and of the health service in supporting this practice.

**Conclusion:**

Consumers want to record their clinical encounters with the consent of their clinician but are unprepared to navigate consent discussions. Health services and clinicians should inform consumers who want to record about their rights and responsibilities, to support the consent process and safe recording environments. Clinician refusal to consent to a patient‐led recording may not lead to increased covert recording; however, clear communication about the reasons for refusing a recording is needed to protect the consumer–clinician relationship.

**Patient or Public Contribution:**

A health consumer was part of the research team and was involved in all stages of this study, including the design, data analysis and reviewing of the manuscript.

## 1 INTRODUCTION

Recording everyday events is synonymous with living in a smartphone society.^1^, ^2^ In high‐resource countries, health consumers are using their smart devices to record aspects of their hospital journey, including encounters with clinicians.^3^, ^4^, ^5^, ^6^, ^7^ When consumers initiate a recording of a clinical encounter, it is known as a patient‐led recording.^8^ Despite a 40‐year history of research which looks at the provision of audio recordings of clinical discussions,^5^, ^9^, ^10^, ^11^, ^12^ the topic of patient‐led recordings is under‐researched. To date, studies on patient‐led recordings have sought to understand the prevalence of recording,^5^, ^7^, ^13^ interest in recording, and motives for consumers to record,^4^, ^7^ the benefits and risks of patient‐led recordings,^3^, ^6^, ^7^, ^14^ as well as enablers for and barriers to integrating recording into practice.^11^, ^12^ Although clinician hesitance to recording is well‐established,^3^, ^6^, ^15^ to date there have been no studies which have explored consumer attitudes and experiences with the consent process, and what happens when a clinician refuses to give consent to the recording. This study sought to further our understanding of consumer attitudes and experiences regarding patient‐led recording, including the consent process, to inform patient‐centred policy and practice.^16^, ^17^

While consumers are initiating recording frequently, the evidence suggests that clinicians are hesitant to accept recording in practice.^3^, ^4^, ^5^ For example, a Canadian study (2019) found that only 27% (*n* = 20/110) of nurses and 30% (*n* = 11/36) of physicians felt that patients should be allowed to take videos within the emergency department.^4^ A recent study of 360 oncologists found that, whilst most were accepting of recording, there were still 25% who reported they were uncomfortable and 15% who never or only selectively allowed a patient‐led recording.^13^ Evidence points to patient‐led recordings being a topic of concern for clinicians,^3^, ^13^ therefore more research is urgently needed to balance the competing needs and interests of consumers and the health service.

In Australia, recording clinical encounters is legislated according to state or territory, except for telehealth/web‐based encounters, which fall under Commonwealth legislation. (Telecommunications (Interception and Access) Act).^18^ The main distinction between these laws is whether the consent of all parties is required.^8^ In Queensland, where this study was undertaken, it is legal for a consumer to make a recording of their clinical encounter without the consent of the clinician. Consumers are legally able to share the recording with family and friends without the clinician's consent.^8^, ^19^, ^20^, ^21^ However, it is an offence for a person to visually record another person in circumstances where a reasonable adult would expect to be afforded privacy,^21^ and recording may be considered an offence if the act of recording is deemed to cause a public nuisance—which may apply in hospital settings.^20^

The hospital policies, where this study took place, instruct clinicians regarding the consent process. They stipulate under what grounds a clinician may choose to give or refuse consent and provide insight into the legislation and clinician rights. Principles include whether the recording will impact on care or health and safety issues; consideration of the privacy of other individuals; restrictions regarding proximity to electro‐medical equipment and within designated places (e.g., not in the operating theatre); and finally, that withdrawal of consent can occur at any time.^22^ Given the complexity surrounding legislation and policy, it is possible that consumers have a limited understanding of their rights and responsibilities with potential implications for practice (e.g., consumers may require guidance from clinicians about where and when they may record). However, with no current research into this issue, it requires further exploration.

In contrast, an audio recording of a clinical encounter that is led by a health service^8^ is an empirically evidenced intervention and has been found to be valued by consumers and linked to positive patient outcomes.^9^, ^10^, ^12^ Whilst there is less evidence which investigates patient‐led recordings, early understandings have emerged around consumer motives for and the benefits of recording. Studies have found the recordings enhance patient understanding, enable shared listening with family and other supports, provide therapeutic benefits, allow for data ownership and evidence of the clinical encounter, increase accountability from the clinician and serve as a memento of a significant experience.^4^, ^5^, ^7^, ^14^ Patient‐led recordings have been found to mirror the benefits of service‐led recordings, which include increased recall and understanding, support for decision‐making and increased patient empowerment.^5^, ^7^, ^9^, ^10^, ^11^, ^12^

Although clinicians recognize some of the benefits of recording,^3^, ^12^, ^13^ clinicians cite several concerns of patient‐led recording, such as confidentiality risks, potential changes to the consumer–clinician dynamic and possible medico‐legal ramifications, which can act as barriers to consenting to the recordings.^3^, ^5^, ^12^ Previous studies have sought to investigate ways that recordings can be integrated into service, including the use of clinical champions^12^ or the use of a smartphone app which aims to mitigate some of the medico‐legal fears held by clinicians.^11^ However, a recent study suggests that these may not necessarily support the integration of recording within the broad spectrum of clinical scenarios in the hospital which consumers wish to record.^3^ Furthermore, clinician refusal of the recording has been linked to increased covert recording.^3^, ^7^ Covert recording is when a consumer makes a recording without the consent or knowledge of the clinician.^8^ Whilst covert recording of clinical encounters is occurring,^7^, ^23^ there is limited research into the consumer perspective on this topic.

There is a dearth of knowledge about the experiences and interests of consumers regarding patient‐led recordings within a broad range of situations and a lack of knowledge about consumer perspectives relating to the associated social exchange, such as the consent process. This has prompted our study, which focuses on answering the research question: what are consumer attitudes and experiences regarding recording a clinical encounter?

## 2 METHODS

To explore this topic, a social constructionist paradigm was used, which maintains that reality is constructed through relationships and interactions with others.^24^ Therefore, to understand a constructed reality, one must explore the meaning and truth that people attach to a phenomena.^24^ The aim of the study was to explore health consumers (patients, their friends, family, or carers) perspectives related to recording their clinical encounters in the hospital. This study was the second stage of a wider, multistage study. The first stage of the study explored *clinician* attitudes to and experiences of patient‐led recording.^3^ This study was conducted at two hospitals within the Gold Coast Health Service, Australia.

Health service consumers were invited to join the research team via the Gold Coast Health Service, Consumer Advisory Group. Only one health consumer responded to the expression of interest and was recruited to the research team. The health consumer was motivated to participate in the research project as the topic was of interest, and she was motivated to develop her research skills. The health consumer had some previous experience working on health‐related projects as a consumer advisor and contributed to all aspects of this study (design, analysis and manuscript). The health consumer is a named author of this paper.

### 2.1 Participants

Participants were patients or family members, friends or carers of patients who were admitted to one of the two hospitals at Gold Coast Health. To participate, consumers needed to be over 18 years old, speak a sufficient level of English to discuss this topic, and be interested in participating in this study. Participants were recruited through advertisements on the health service's social media sites and on digital screens around the hospital. Paper flyers were disseminated on ward receptions and in hospital waiting areas (including outpatient clinics). Also, invitation letters were handed out by a member of the research team to people in the hospital common areas and to patients admitted on the wards. If prospective participants were interested in learning more about the project, they were provided with a participant information and consent form. This advised them of who was conducting the study, the aims of the study, what the study would involve, confidentiality, and their options to withdraw. All participants who expressed interest in the study were eligible to participate. Of the participants who expressed interest in the study, one declined to be involved. Participants were advised there would be no negative consequences as a result of participating or withdrawing from the study. Participants provided written and verbal consent. Participants were also invited to write their gender and age on the participant information and consent form.

### 2.2 Interviews

Twenty participants were recruited for the study. Twenty participants were selected to allow for novel and diverse data regarding the phenomenon. The group was also small enough so that the topic could be explored deeply.^25^ Semistructured interviews of approximately 45 min were conducted with each participant. Semistructured interviews were selected as they allowed participants the opportunity to have some control of the dialogue, whilst ensuring that the data retrieved was relevant to the research questions.^26^ Participants were offered interviews in person at the hospital or via video conference. For participants who chose to be interviewed in person, they were offered a private meeting room in the hospital. Participants were not paid for the interview; however, the cost of parking at the hospital was offered.

The team developed a semistructured interview guide with questions and prompts (see Supporting Information). The guide was internally piloted before being used with participants. A health consumer representative was also on the research team and was able to provide a consumer perspective when developing the interview guide. Interviews were conducted by a member of the research team (a female, clinical social worker) who was experienced in qualitative research and interview techniques. Interviews were digitally audio recorded and transcribed (intelligent verbatim). The interviewer completed a reflective journal to enhance the quality and integrity of this study.^27^ Participants were given the option to review the transcripts for accuracy before analysis. There were no major changes made by participants who chose to review their transcripts.

### 2.3 Data analysis

Interview transcripts were uploaded to NVivo to support the analysis process. Inductive thematic analysis was used, applying Braun and Clarke's^28^ six‐phase process. This methodology was selected as it allows for both a reflection and deconstruction of participant realities.^28^ Two researchers separately orientated themselves with the data and identified codes based on patterned or significant responses, ensuring all relevant data were represented. Next, they collated these codes into potential themes. Then, the same two researchers collaborated to further refine themes, reconciling discrepancies and defining and naming themes. Quotes were selected to represent key themes. Finally, a report was generated and sent to the rest of the research team for final analysis.^28^, ^29^

## 3 FINDINGS

We interviewed 20 participants, who provided their gender and age information on the participant information and consent form. A total of 11 participants identified as male and nine as female. Participant ages ranged from 25 to 68 years old, although one participant did not state their age. Most participants were current or recent Gold Coast Health patients (inpatients and outpatients). Three participants were the family of patients.

Four main themes were identified relating to the patient‐led recording of a clinical encounter: (1) consumers recognized and acknowledged the importance of obtaining clinician consent, (2) clinician refusal of recording had the potential to undermine the consumer–clinician relationship, (3) that consumers were uninformed and misinformed about hospital policy and legislation and (4) consumers hold expectations regarding their rights to record and the role of the hospital in facilitating recordings (Table 1).

**Table 1:** Themes and codes

Themes	Codes
Theme 1: Importance of consent	− Strong motives to obtain consent from clinicians. − Inconsistent accounts of the consent process. − No previous covert recording behaviour reported. − Limited anticipated covert recording behaviour in future. − Covert recording mainly linked to poor experience of health service.
Theme 2: Refusal may undermine the relationship	− Clinicians should consent to the recording if important to consumers. − Refusal to record requires a rational. − Refusal linked to questioning clinician integrity or competence. − Preference for a clinician who was open to recording.
Theme 3: Uninformed and misinformed	− Uncertainty about policy, legislation and rights. − Lack of accurate information provided by hospital.
Theme 4: Consumer expectations	− Defending the right to record. − Consumer sense of ownership of the content the clinical encounter. − Hospital role to inform consumers about their rights and hospital rules. − Lack of consensus regarding formats of information provision. − Clinicians should offer recording.

### 3.1 The importance of consent

Many participants reported previously recording their hospital clinical encounters, although a few had not. Of the participants who had recorded, a few had recorded once or a few times and some had recorded multiple times throughout their admission or health service experience. Participants gave examples of recording discussions with doctors and nurses, therapy sessions with allied health clinicians and recording brief clinical encounters during their admission, such as receiving nursing care.

Most participants felt obtaining clinician consent before recording was important. ‘It needs to be consensual on both parties … So it needs to be asked before done’ (P1). All the participants who had previously recorded, advised that they had done so with the consent of the clinician. However, consent was understood differently by different participants. Some participants had verbally asked for consent from the clinician before initiating the recording. ‘They were fine with it. But they were just like, as long as you don't get our complete face in the video, that's fine’ (P3). For others, they believed consent was obtained, despite no formal discussion occurring. ‘Well, they could see we were doing it, so we didn't actually ask permission … They seemed more than happy and understanding’ (P5).

Participants were motivated to obtain consent to meet their understanding of the legal and policy requirements, or because they viewed it as morally right or the courteous course of action. ‘I just think it's nice just to ask, because I'd want somebody to ask me if they were going to record me’ (P9). For some participants, whilst obtaining consent was viewed as important, it raised other concerns, such as whether asking to record would negatively affect the consumer–clinician relationship. Participants also considered how the request would impact the clinician. This deterred participants from initiating a recording. ‘I just wouldn't want to put someone [a clinician] in the position where they had to say no to it or they had to go along with it’ (P12).

None of the participants reported undertaking a covert recording (recording without the consent or knowledge of the clinician). Most participants were against covert recording and believed it was illegal, disrespectful or raised concerns about the impact on their care if the recording was discovered. ‘Yeah, that's illegal. I couldn't do that to somebody because then if that came up, I could be out of this hospital and the treatment would get worse’ (P11). Some participants viewed covert recording as problematic as they identified with the clinician's position and expressed their own wishes not to be recorded covertly. ‘Just because I don't want to be recorded without my consent, so I'd feel hypocritical if I did’ (P12).

Most participants reported they did not anticipate they would ever consider recording covertly. However, there were a few participants who acknowledged that they might record without the consent of the clinician in the future. The main reason for recording without consent was to obtain evidence of a poor experience of the health service that could be used for complaint procedures or to garner support. Poor experiences included unpleasant interactions with clinicians and operational inadequacies.

It would feel weird doing it, but I remember me and my partner at the time were thinking what is going on? This is a bit – this is rude. This isn't the normal conversation you'd have with someone unless they're at the end of their day and they're really kind of over it. It would be nice to be able to show someone else and go it wasn't just my point of view. (P8)

One participant reflected on a difficult clinical encounter where she had felt unheard by the treating team. The participant believed that a recording would have empowered her by providing evidence of the service she received. The participant indicated she might record without consent as she didn't think the clinician would give consent to this recording. ‘Well, I'm sure they would have said no, so no, I would have not even asked them’ (P2). Another participant did not think consent was required and mentioned that they might record sections of their admission without the consent or knowledge of clinicians. ‘I don't see no harm in just leaving the camera there rolling at times. I'm not going to do it all the time, but some days I might think, oh yeah, put the camera there’ (P14).

### 3.2 Refusal may undermine the relationship

Participants reflected on how a clinician's refusal or stopping of the recording would affect the consumer–clinician relationship. Despite expressing understanding of the clinician's position not to record, most felt that refusal could negatively affect the relationship. This depended upon the reasons given by the clinician and the context in which the recording had been requested. There was a view that the clinician preference (not to be recorded) should not supersede important consumer needs.

I felt like I needed to for my safety or so on, then obviously I would feel very uncomfortable … But just for the fact to say I was recording to show a friend at home or something and they didn't want me to, then that's fair enough. (P20)

Another participant reflected on how the clinician was not equipped to assess the potential value of the recording to the consumer, therefore should not refuse or stop a recording.

But you can never really judge that—why they're recording. No, I don't—I'm trying to think of a reason why you'd want to cancel or say please stop recording me unless it was getting heated or there was some type of disagreement on something. But you know you come to a GP or you come to a hospital expecting information. So no, I don't see—I do not see why you would stop recording it or be asked to stop recording it. (P12)

For some participants, clinician refusal signified a lack of transparency in the service and aroused or catalysed concerns about the integrity and competence of the clinicians. ‘What are you hiding from me? Because I know they only give you a certain amount of information. They don't give you everything, but why would they not want to be recorded?’ (P9). Participants felt this had the potential to lead to a loss of trust in the clinician and a breakdown of the relationship. ‘I would probably not trust, yeah, I think it would be a little bit of a loss of trust. Maybe the clinician doesn't know much or he's not keen to help’ (P2).

There were few participants who expressed frustration regarding previous experiences of being refused or asked to cease a recording. ‘She [the nurse] come in and told me that it was a privacy breach, that I wasn't allowed to film in the hospital at all and basically just shut it down. She was really mad’ (P13). Some participants felt they were entitled to receive care from a clinician who was willing to be recorded and would request another clinician in the event the recording was refused: ‘Then they can leave the room because that means there's something that's not being done right. No offence’ (P11).

Some participants felt uncertain about how to balance clinician needs with maintaining the consumer–clinician relationship. ‘Look, I respect their rights and their choices. But also, it makes you think, what do you have to hide?’ (P1). Some participants were unaffected by a clinician refusing or stopping a recording and reported it had not negatively impacted the consumer–clinician relationship. This was linked to previously reported understandings of clinician reasons for refusal or the participant's own disinterest in recording.

### 3.3 Uninformed and misinformed regarding policy and legislation

Participants were uncertain of the legality or hospital policy as it relates to the consent process when recording. Multiple participants held false views about the legislation and policy of recording in hospitals. Some falsely believed that recording a clinical encounter was either illegal or not permitted under the hospital policy. They reported that this had prevented them from initiating a recording. ‘That's probably one of the other reasons that I would never record anything because I'm not actually aware of any of these practices that may happen in a hospital’ (P6).

Many participants incorrectly thought it was illegal to make a recording without the clinician's consent. Some participants had been falsely informed by hospital staff that recording was not allowed or had misconstrued signs asking consumers not to record in public areas. A few participants understood their rights to record although were uncertain about the hospital policy. One participant felt that whilst recordings were legal, they were discouraged by the health service.

I believe I'm allowed as we're in a public health system, and I believe that being a member of that public health system they can't really say yes or no. I just believe they don't like you doing it because it gives a bad rep to the hospital if something comes out of it and they lose funding and they lose all the rest of it and everyone goes, oh the Gold Coast Hospital sucks. (P13)

### 3.4 Consumer expectations

Despite being uncertain about their rights, almost all the participants felt that consumers should have the right to record their clinical encounters. Some participants gave the caveat of depending on both parties consenting and the circumstance surrounding the recording. Some participants held strong views about their rights to record and linked this to ownership of the clinical encounter. One participant defended their right to record by highlighting perceived inconsistencies in the way that smartphones were used to record and share health information when it benefited the health service.

Very offended because, sorry but it's none of your business. It's my private health, and if I choose to share it, I'm allowed to do that, especially in this day and age where we're sharing vaccine reports and everything. (P13)

There was one participant who was quite opposed to recordings generally. ‘I'd hate to see a day when you came into a hospital and there was video recorders from all different angles and people—the patients were recording, the nurses were recording, and we'd actually forgotten about treating people or other people were getting left out of the loop as far as treatment and attention goes’ (P16). However, he still felt that consumers should have the right to record. ‘I mean, it goes both ways, doesn't it? … No, every individual has the right to record or right or whatever, but they have to have the approval prior’ (P16).

In contrast, some participants were less concerned about the right to record. These participants had not previously felt the need to record a clinical encounter, were sceptical about the benefits of recording, or were happy with the current state of healthcare delivery and did not consider recordings necessary. ‘No. No, I'm pretty good either way. I don't need to record it’ (P19).

Most participants were motivated to receive more information about their rights and legal matters about recording in the hospital. Some felt strongly that the hospital had a role in proactively informing consumers about their rights to record.

Definitely needs to be some kind of a brochure given to you, yeah, when you are in hospital, like probably as soon as possible. So, you know if you wanted to do it, you can do it. If you don't want, that's fine. (P2)

Some participants did not consider that further information relating to patient‐led recording was required, as they either believed they knew the legislation and policy or were uninterested in learning more. There were many ideas about how information about legislation and policy could be disseminated to consumers, including brochures, posters, information on the website, verbally provided by the clinician when a consumer initiated a recording or during the admission process, and in the hospital information kit.

Recording was viewed by many to be a useful tool with several perceived health engagement, psycho‐social and service‐navigation benefits. Many participants felt that clinicians should take control of the consent process by encouraging or offering consumers the option of recording. ‘Maybe they could if they'd accept it, they could offer it, say if you want to record this you can’ (P15). Offering a recording was viewed as a solution by participants who didn't think to record amid a clinical encounter ‘They should have offered—and especially—we've had meetings with five or six doctors and they're all saying different things at the same time’ (P7). Clinicians offering recordings were also seen as overcoming physical barriers to recording. ‘Being limited with hands, help, I need help to turn it on, because I do get restricted, like turning it on’ (P10). For those with strong views in support of recording, they suggested the clinician played an important role in enabling consumers to access this tool. ‘Encourage it. Let people know you're allowed to do it. Doesn't hurt to take a photo. Doesn't hurt to record’ (P11).

## 4 DISCUSSION

This study sought to understand health service consumer perspectives and attitudes regarding patient‐led recordings in hospitals. Four main themes were identified which contribute to knowledge of this topic. First, most participants viewed obtaining consent from the clinician as important and, whilst it was considered an option by some participants, covert recording was not generally supported. Second, that a clinician refusing a recording had the potential to undermine the relationship, leading participants to question the clinician's integrity and competence. Third, we found that participants were mostly ignorant of policy and legislation or were uninformed or misinformed. Finally, participants had expectations both about their rights to record and also the role of the hospital in providing relevant information and in supporting the integration of recording in practice.

Clinician refusal of a consumer's request to record has been linked to increased covert recording and relationship conflict.^3^, ^7^, ^12^, ^30^, ^31^ Covert recording is a stressful experience for clinicians who worry about the professional and medico‐legal consequences.^3^, ^23^, ^31^ Our findings indicate that covert recording is generally not appealing to consumers. None of the participants reported previously making a covert recording and most were against the idea of covertly recording in the future. Our findings indicate that consumers hold a range of beliefs and values which means obtaining clinician consent before recording is important. Participants were able to identify with the clinicians and were motivated to maintain transparent relationships, which met the interests and needs of both parties. These findings may provide some reassurance to clinicians who are fearful of covert recording^23^, ^32^ in a world dominated by smartphones.^1^, ^2^

There were some findings from our study which are cause for concern for clinicians and health services. As previously reported in the literature, our study found some participants would consider covertly recording in the future, due to poor experiences of the service and the need to obtain evidence to support their claim.^7^, ^23^ This behaviour suggests long‐understood power imbalances within the consumer–clinician relationship may persist,^33^, ^34^, ^35^ with indicators that the usual feedback or complaints mechanisms are not adequately meeting consumer needs. Whilst work has been undertaken to address the asymmetrical relationship through patient‐centred care and patient empowerment strategies,^36^, ^37^, ^38^, ^39^ consumers may continue to hold beliefs that their experiences will not be believed or complaints actioned. If health services truly wish to implement an espoused person‐centred care approach, then our study suggests there is still considerable work to be done in establishing and maintaining trusted and collaborative partnerships with consumers.

Significantly, findings from this study show that the partnership between clinician and consumer is jeopardised when a clinician refuses a recording. Whilst previous studies have alluded to this,^3^, ^5^, ^23^, ^31^ our study is the first to show the range of detrimental effects that a clinician refusing a recording may have on the relationship. Responses spanned questions regarding clinician integrity and competence to the complete breakdown of the relationship. One of the most valuable findings from this study is that the relationship might be salvaged if clinicians provide a clear rationale for refusal, particularly if reasons for refusal are not of a personal nature. Our findings indicate that some consumers are not understanding of the multiple reasons why a clinician might refuse or ask them to cease recording, such as potential risks to patient care and safety, impact on the clinician–clinician rapport and confidentiality reasons.^3^, ^4^, ^6^ Educating consumers about these risks is important to preserve the consumer–clinician relationship. However, prioritizing the integrity of the relationship is an important consideration in the current climate of distrust in the healthcare sector.^40^, ^41^, ^42^ Clinicians who regularly refuse recordings may need to both re‐examine their practice, consult hospital guidelines as to consumer–clinician rights and consider how to mindfully communicate a refusal if that is still their preference.^3^, ^6^, ^13^

Our findings revealed that consumers are not aware of their rights or responsibilities when it comes to recording a clinical encounter. This is both a barrier to them using a potentially beneficial tool and a reason why they may not verbally seek consent, with the potential to lead to more covert recording. In addition, whilst consumers reported they were keen to obtain consent, there was uncertainty and inconstancy about what constitutes consent. If a consumer starts recording and the clinician does not object, is this really consent? Findings from a previous study (2022) into clinicians' attitudes to and experiences of patient‐led recordings infer that some clinicians may not feel they had the option to refuse a recording. Further findings were that some clinicians experience stress when participating in discussions about recordings whilst completing their work, with potential risks to patient safety.^3^ Ensuring both consumers and clinicians share understandings of the consent process may be key in building safe recording and working environments.^11^

Educating clinicians about the relevant hospital policy and legislation may be central to meeting consumer expectations surrounding recording clinical encounters. First, our findings suggest consumers believe clinicians and health services should be responsible for informing them about their rights and responsibilities to record. However, since evidence indicates that the clinicians are themselves uncertain about the policy and legislation, there is a need to improve clinician knowledge to meet consumer requirements.^3^, ^6^, ^13^ Second, our findings show that some consumers want clinicians to take control of this process and offer recordings. Whilst evidence generally supports clinicians offering consumers the option to record,^7^, ^10^, ^12^, ^30^, ^43^ this is not common practice.^3^, ^6^, ^12^ The literature suggests that increasing clinician confidence in discussing hospital policy, legislation and the consent process may lead to greater acceptance of recording and control over this process.^11^, ^12^ Regardless of who initiates the recording, both consumers and clinicians need to be educated about the legislation and policy so that they can engage in open discussions about the benefits and risks of proceeding with a recording.

### 4.1 Limitations and strengths

The findings of any study should be considered alongside its' ontological assumptions, strengths and limitations. This is a small qualitative study within a constructionist paradigm, which rejects the notion of objective reality. In accordance with the ontological perspective and research design, generalisable data were not sought. Instead, the study aimed to illuminate constructed realities, which was achieved. However, there were several weaknesses which may affect the quality and trustworthiness of these findings.

Participants were made aware that their health service was conducting this study. Also, whilst participants were offered a meeting room or video conference, some of the participants opted for interviews to be conducted at their bedside, during their hospital admission. Both study characteristics could have led to bias in participant responses. For example, participants may have been influenced by privacy concerns, or fear about possible repercussions of reporting certain thinking or behaviours (especially in relation to covert recording). Further, responses may have been affected by health acuity. On the other hand, discussing hospital experiences with participants whilst they were admitted may have improved the quality of the findings. Participants were able to discuss recent experiences and consider the questions about the health service from within the environment. Interviewing participants from other age groups may have led to more diverse understandings of this topic. Also, we only interviewed participants who were proficient in English, which again may have impacted the quality of the findings. Particularly as there is growing evidence which shows the benefits of audio recordings of clinical discussions for this population.^44^

A strength of this study was the use of semistructured interviews, which allowed participants to discuss and expand on items not considered by the research team. This enriched and broadened the data retrieved. Another important strength of this study is the composition of the research team, which was made up of academics, a clinician, a member of hospital management and a health consumer. Involving a health consumer throughout the research life cycle enhanced both the quality and relevance of the findings via the lived experience.^45^, ^46^

## 5 CONCLUSION

Patient‐led recordings of clinical encounters are more commonly part of the patient's health journey and are a beneficial tool for patient care. Consumers are motivated to obtain consent from clinicians to record, although are not well equipped to navigate this process, due to poor knowledge of their rights and responsibilities. In response, health services need to make information available to consumers who want to record. Clinicians should understand the relevant legislation and hospital policy. They also need to carefully consider their reasons for refusing a recording and provide quality communication for consumers as a relationship imperative.

## AUTHOR CONTRIBUTIONS

Laura Ryan and Robyne Le Brocque conceptualized the study. Laura Ryan, Robyne Le Brocque, Kelly Weir, Jessica Maskell and Lily Bevan designed the study. Laura Ryan collected the data. Laura Ryan and Robyne Le Brocque analysed the data. Laura Ryan, Robyne Le Brocque, Kelly Weir, Jessica Maskell and Lily Bevan contributed to the interpretation of the data. Laura Ryan drafted the manuscript with input from Robyne Le Brocque, Kelly Weir, Jessica Maskell and Lily Bevan. All authors gave approval for the final version of the manuscript.

## ACKNOWLEDGEMENT

We acknowledge and pay respects to the people of the Yugambeh language region of the Gold Coast and all their descendents both past and present. We also acknowledge the many Aboriginal people from other regions, as well as the Torres Strait and South Sea Islander people who now live in the local area and have made an important contribution to the community. We wish to thank the health consumers who shared their experiences with us, and Joanne Hilder, who provided support thoughout this project.

## CONFLICT OF INTERESTS

The authors declare that there are no conflict of interests.

## Supporting information

Supporting information.

Supporting information.

## Data Availability

Data available on request due to privacy/ethical restrictions
