# Supplementary material for: ‘What are you hiding from me?’ A qualitative study exploring health consumer attitudes and experiences regarding the patient‐led recording of a hospital clinical encounter
Source: Health Expect. 2022 Oct 13;25(6):3096–104. doi: 10.1111/hex.13617 (PMC9700167; doi:10.1111/hex.13617)
Supplement: Supplementary file 1 — Supporting information. [file HEX-25--s001.docx]

**Interview Guide**

| **When Patients Record Their Clinical Encounters: A Qualitative Study (Stage 2)** |  |
| --- | --- |

**Introduction**

Thank you for agreeing to be interviewed for our ‘Recording Encounters 2 Study’. We are undertaking this study to explore the attitudes and experiences of patients recording their health journeys using a smart device (audio or video recordings) at Gold Coast Health.

As outlined in the Participant Information and Consent Form, this interview will be digitally audio recorded, de-identified and then sent for transcription by an approved transcription service, after which we will undertake qualitative analysis. This interview may take 45 - 60 minutes.

Just to confirm, all information is confidential and any publications arising from this study will ensure that individuals will not be able to be identified, and that you have the right to withdraw from the study at any time. If you are happy to proceed, we will commence the interview.

*Experiences with recording clinical encounters*

1. **Could you describe any experiences where you or a support person attempted to record or recorded a clinical encounter? A clinical encounter is any contact with a health care worker, such as a meeting with a doctor, a nurse helping a patient to get dressed in hospital, a physio providing therapy or a family meeting with multiple health care workers. Please take some time to think about this experience. Start at the beginning and describe what occurred and how it made you feel.**

Prompts:

- In what kind of setting did this occur?
- What made you want to record?
- Did you ask permission? If so, who to and how was this discussion for you?
- How was the recording stored?
- How was the recording used?
- Where is it now?
- Did it achieve its desired benefits?
- Were you satisfied with the way that GCH clinicians communicated with you?
- Was it a positive or negative experience?

1. **How did the health care worker react to your recording or asking to record the encounter? Do you have any thoughts on why they reacted that way?**
2. **Is recording a clinical encounter something you do usually?**

Prompts:

- Why/why not?
- What supports or barriers to recording are you aware of?
- Would you usually ask permission and how and who would you ask?

1. **Would you like to record more of your clinical encounters?**

Prompts:

- - Why/ why not?
  - What would help to facilitate those?
  - Do you think this could change in the future?

1. **Have you had an experience where a health care worker offered or initiated a recording?**

Prompts:

- - How did this make you feel?
  - Was it a positive or negative experience?
  - Do you think clinicians should offer to record clinical encounters more/less/same?
  - How should recordings be offered/ initiated with patients?

1. **Just a reminder that this interview is confidential. Can you describe a situation where you either have recorded or might consider recording a clinical encounter without the knowledge and/or consent of the health care worker?**

Prompts:

- - In what context did this happen? (Place, practice area, activity)
  - What led to you wanting to record?
  - What were the reasons you didn’t ask permission?
  - How did you feel about this?
  - What were the outcomes of having the clinical encounter recorded?
  - Were you satisfied with the way that GCH clinicians communicated with you?

*Patient perspective*

1. **What is your perspective or attitude to recording clinical encounters?**

Prompts:

- - How does recording impact on your experience of the health service or interactions with health care workers?
  - Have you had any negative experiences with recording?
  - Is there something you would not like recorded?
  - Do you have strong feelings on this?
  - Do you think there are times when a patient or their family member should record the clinical encounter?’

1. **How would you feel if a health care worker refused for the encounter to be recorded?**
2. **In your opinion, what are the benefits of recording your clinical encounters?**
3. **In your opinion, what are the risks or issues of recording your clinical encounters?**

Prompts:

- What do you think are the confidentiality risks of making a recording?

*Awareness of policy and legal issues*

1. **Can you describe your understanding of the hospital policy and your rights or legal considerations when it comes to recording your clinical encounters at GCH?**

Prompts:

- - Do you think patients should have the right to record their clinical encounters?
  - Do you think GCH should be able to ask patients to stop recording? In what scenario?

1. **Can you describe the rights or legal considerations of other people in the clinical encounter (such as doctor, nurses, health care professionals) if you would like to record the encounter?**
2. **Do you think GCH provides enough information about patient recordings?**

Prompts:

- What further information would you like?
- How would you like this disseminated/provided?
- Is there anything further GCH clinicians can do to support you in this space?

*Wrap up*

1. **Do you have any other thoughts or points of interest you would like to add? (Interviewee or interviewer)**

*Thank you so much for participating in our research.*

**Interview completed.**
